# A Review of Infant and Young Child Feeding Practices and Their Challenges in India

**DOI:** 10.7759/cureus.66499

**Published:** 2024-08-09

**Authors:** Mayank Sharma, Abhay Gaidhane, Sonali G Choudhari

**Affiliations:** 1 Community Medicine, Jawaharlal Nehru Medical College, Datta Meghe Institute of Higher Education and Research, Wardha, IND

**Keywords:** india, malnutrition, nutrition, complementary foods, exclusive breastfeeding, infant and young child feeding (iycf)

## Abstract

This review focuses on infant and young child feeding (IYCF) practices in India, aiming to offer information on its trends, challenges, and opportunities for improvement. The overview starts by exploring the importance of IYCF practices and their results on child health, growth, and development. It delves into cultural norms, conventional practices, and local variations that impact feeding behaviors, acknowledging the range of nutritional habits across communities. The role of healthcare systems and community interventions in promoting the most desirable feeding practices is mentioned, addressing issues consisting of different breastfeeding practices, well-timed introduction of complementary meals, and micronutrient supplementation. By making this assessment, the goal of this review is to make healthcare professionals, policymakers, and researchers aware of the current trends of IYCF and its demanding situations, and regions for development in India. It gives an understanding of the improvement of strategies and interventions that can make contributions to the increase and improvement of infant and young child nutrients, thereby nurturing the Upcoming generations.

## Introduction and background

The first 1,000 days, from birth to two years, are crucial in ensuring optimal growth and development. Infant and young child feeding (IYCF) practices play a vital role in the growth and development of the youngest contributors to society. The World Health Organization (WHO) recognizes the significance of early nutrition and recommends exclusive breastfeeding (EBF), which means feeding infants only breast milk for the first six months. After six months, introducing complementary feeds (CF) means introducing solid or semisolid foods alongside breast milk until two years of age. The WHO has partnered with the United Nations International Children's Emergency Fund (UNICEF) to promote these feeding practices and create a policy known as IYCF. Additionally, they have established population-based metrics to assess these practices [1].

Globally, nearly half (45%) of mortalities in children below the age of five are linked to malnutrition, with the majority of these mortalities, almost two-thirds, occurring in infants as a result of insufficient dietary intake [2]. India is currently a home for over 33.33% of the malnourished children [2]. As per National Family Health Survey-5 (NFHS-5) data, there has been a marginal rise in the proportion of children experiencing severe malnutrition and those who are overweight. However, the concerning trend is the significant 8% point increase in the old and new cases of childhood anemia, which escalated from 59% in the National Family Health Survey-4 (NFHS-4) to 67% in NFHS-5 [3]. The cases of small heights for age have given indications of concern among nutrition experts in the public health field regarding the development of children [4]. Persistent malnutrition continues to be a notable challenge in South Asian areas, primarily stemming from limited access to nourishing food and a lack of sufficient information or education to enable informed dietary decisions [5]. The significant repercussions of malnutrition extend to social and economic aspects, encompassing effects such as compromised cognitive development, the onset of chronic diseases, and diminished future income potential [6].

The National Guidelines on IYCF 2019-20 by the Indian government focuses on early initiation of breastfeeding, EBF, for the first six months of life for optimum nutrition. After six months, as their dietary needs evolve, infants must be regularly added to nutritionally suitable and safe CF while continuing breastfeeding for a minimum of two years or even longer [7]. These IYCF guidelines guarantee that infants acquire the optimum nutrition during the early two years of life [8]. Insufficient complementary feeding practices and unhygienic habits can result in recurring infections, malnutrition, growth impediments, immunodeficiency, and, in extreme cases, fatal consequences [9]. The National Guidelines on IYCF cover a variety of factors, including dietary practices, caregiving practices, hygiene protocols, and social connections. Implementation of these guidelines necessitates support at various levels, including the child, mother, household, community, and society [7,10]. Enforcing proper IYCF practices at the grassroots level, utilizing established networks of Anganwadi workers, auxiliary nurses and midwives, and accredited social health activist workers to educate mothers, has the potential to substantially decrease infant mortality rates, especially in rural regions [11].

## Review

Defining IYCF

IYCF is a set of well-known and common recommendations for appropriately feeding newborns and children under two years of age. It includes early initiation of breastfeeding, EBF for the first six months, and complementary feeding after six months [1].

Early initiation of breastfeeding refers to starting to breastfeed as soon as possible after birth, ideally within the first hour, for all normal newborns, including those delivered via cesarean section. Since colostrum, the milk secreted during the first two to three days of life has a high concentration of immune-system-boosting cells and immunoglobulins, it should be fed to the newborn instead of being thrown out. The newborn should not be given any prelacteal fluid [1,4].

EBF means that during the first six months of life, infants should only be breastfed by their mother, a wet nurse, or expressed breast milk; they should not be given any other liquids, solids, or even water. The only exceptions are when oral rehydration solutions, oral vaccinations, vitamin and mineral supplements, or medications are administered [11].

When a child reaches the age of six months, complementary feeding refers to adding breast milk to solid or semisolid food. Breast milk is no longer adequate to meet an infant's nutritional needs after six months of life. Nonetheless, newborns are susceptible when they go from exclusively breastmilking to receiving additional feedings beyond breastmilk. Breastfeeding must continue along with suitable supplemental feeding to guarantee that a young child's nutritional needs are satisfied. It is appropriate to use the term "complementary feeding" rather than "weaning" [1,11].

Benefits of optimal IYCF

It is now acknowledged that one of the most successful strategies for ensuring a child's survival is early and EBF. This is especially true when it comes to reducing morbidity and mortality associated with three major conditions: pneumonia, diarrhea, and newborn infections [1,9].

Scientific evidence shows that early breastfeeding initiation significantly lowers neonatal mortality. Until six months, EBF can avert up to 13% of the projected mortality rate for children under five. Additionally, breastfeeding fosters ongoing, healthy interactions between mother and child, which can support a baby's emotional and psychological growth. It has been discovered to benefit brain development directly [4,9].

Breastfeeding contributes to long-term benefits by preventing childhood obesity and lowering the risk of various chronic conditions, such as diabetes, heart disease, and asthma, in adulthood. While breastfeeding gives the child the best nutrition possible and guards against infections, age-appropriate supplemental feeding can significantly lower the burden of disease associated with stunting by starting at the right time. Every child's first 1,000 days are crucial and are considered a window of opportunity for survival and development [1,4].

Investing in nutrition during this time is crucial because stunting caused by undernutrition during this period is almost always irreversible. Achieving children's growth potential through ideal infant and early child feeding during the first 1,000 days is the solution to preventing stunting. According to estimates, an adequate supplementary feeding program can reduce rates of morbidity and malnourishment and prevent 6% of under-five deaths [4,9].

Growth, health, and development

Sufficient nourishment during the early stages of life guarantees children's optimal growth, well-being, and development. Childhood obesity represents a growing public health concern in many nations and is a result of inadequate nutrition. There is a connection between long-term growth, health impairment, and early nutritional deficiencies. Stunting, which results from malnutrition during the first two years of life, causes an adult to be several centimeters shorter than their potential height. There is proof that adults who experienced early childhood malnutrition perform less intellectually. They might also be less able to perform manual labor. Through optimal feeding, the first two years of life offer a crucial window of opportunity for ensuring children's appropriate growth and development. Achieving universal coverage of optimal breastfeeding could avert 13% of deaths in children under the age of five worldwide, according to evidence of the effectiveness of interventions. Appropriate complementary feeding practices would also reduce under-five mortality by 6% [1,9].

The global strategy for IYCF

In 2002, UNICEF and WHO adopted the Global Strategy for IYCF. The plan was created to raise awareness of the effects that feeding habits have on infants' and young children's survival, growth, and development, as well as their nutritional status. It is imperative that every health professional possess the Global Strategy's tenets to protect, promote, and support appropriate IYCF [1].

Status of IYCF globally

There is a general lack of good breastfeeding and supplemental feeding practices. Only 34.8% of babies worldwide are thought to be breastfed exclusively for the first six months of their lives; most babies receive some other food or fluid during this time. Complementary foods are frequently unsafe and of inadequate nutrition, and they are frequently introduced either too early or too late. Sixty-nine percent of births in the developing world are represented by data from 64 countries, suggesting that things have improved in this regard. The percentage of babies exclusively breastfed for their first six months of life rose from 33% to 37% between 1996 and 2006. Sub-Saharan Africa saw significant increases, with rates rising from 22% to 30%, and Europe saw increases from 10% to 19%. With Brazil and Mexico excluded, the percentage of babies in Latin America and the Caribbean who are exclusively breastfed climbed from 30% in 1996 to 45% in 2006 [9].

IYCF trends in India

The IYCF policy instituted by the government of India strongly advocates for EBF during the initial six months as a vital measure to guarantee prime health and growth with the development of the young population of children [9]. As infants progress in age, the requirement for safe and nutritious CF becomes essential. A well-rounded diet for optimal nutrition should incorporate a variety of food groups, including roots, grains, nuts, legumes, and dairy products like yogurt, milk, and cheese. In addition, it should include flesh foods such as fish, meat, poultry, organ meats, eggs, vitamin A-rich fruits, and vegetables. It is crucial to introduce these foods while breastfeeding for a minimum duration of two years [10]. To encourage IYCF practices in India, the government has introduced various health services and schemes (Table 1). The objectives of these services are to create awareness about the importance of IYCF, with the ultimate goal of achieving optimal feeding practices [7].

**Table 1:** Actions taken by the Government of India to promote IYCF MAA: Mother Absolute Affection; IYCF: infant and young child feeding Source: [11] These are open-access data and distributed under a Creative Commons license

Provisions	Actions implemented
Anganwadi Services Scheme	Provides infant feeding counseling to pregnant and lactating mothers
Prime Minister's Overarching Scheme for Holistic Nourishment Abhiyaan	Dominates the optimal infant and child feeding practices and addresses malnutrition through behavioral change communication
MAA	Promotes complementary feeding practices with the Ministry of Women and Child Development
Ministry of Women and Child Development collaborates with MAA	To support vulnerable women and young kids
Home-Based Care for Young Children is an extension of Home-Based Newborn Care	Provides community-based care by accredited social health activists
Village Health Sanitation and Nutrition Days	Provide maternal and child health services and promote awareness of maternal and child care
Mother and Child Protection Card, a joint initiative by the Ministries of Health and Family Welfare and Woman and Child Development	Counseling tool for frontline workers to improve IYCF practices in children

Key findings of infant feeding practices in India from CNNS

Key findings of IYCF practices in India from the Comprehensive National Nutrition Survey (CNNS) (2016-18) include the following: 57% of children aged 0-24 months were breastfed within one hour of birth, while EBF was seen in 58% of infants under age six months. Eighty-three percent of children aged 12-15 months were still breastfeeding at one year of age, while complementary feeding was initiated for 53% of infants aged 6-8 months. Regarding minimum dietary diversity, meal frequency, and acceptable diet, 42% of children aged 6-23 months were fed the minimum number of times per day for their age, 21% were fed an adequately diverse diet, and 6% received a minimum acceptable diet. Among school-age children and adolescents, more than 85% consumed dark green leafy vegetables and pulses or beans at least once per week, while one-third consumed eggs, fish or chicken, or meat at least once per week, and 60% consumed milk or curd at least once per week [12].

NFHS survey findings and trends

The NFHS-5 (2019-21) data show improvement in breastfeeding initiation practices, with a notable increase in the fractions of infants being breastfed within one hour of birth compared to earlier survey findings. The data from previous NFHS surveys (4, 3, and 2) also indicate an upward trend in breastfeeding initiation, showcasing a consistent pattern of improvement over time. There has been significant improvement in EBF for the first six months compared to the data from NFHS-4. Previous NFHS surveys (4, 3, and 2) consistently reported low trends in EBF, indicating persistent challenges in achieving optimal breastfeeding practices. NFHS-5 survey reveals a positive trend in the introduction of CF aged 6-8 months. The previous surveys of NFHS 4, 3, and 2 also indicated a growing trend in well-timed complementary feeding, signaling a gradual shift toward improved feeding practices. The recent survey underscores a remarkable improvement in diversity in the diet among children in the age group of 6-23 months. This significant change indicates improved access to various nutritious foods, contributing to better nutrition. Previous NFHS surveys (4, 3, and 2) also reported improvements in dietary diversity, although the progress varies across the surveys [13,14].

Table 2 shows the trends of major indicators of IYCF practices in India according to NFHS 2, 3, 4, and 5.

**Table 2:** Trends of major indicators of IYCF practices in India NFHS: National Family Health Survey; IYCF: infant and young child feeding Adapted from: [11,12] These are open-access data and distributed under a Creative Commons license

Indicator	NFHS-2 (1998-99)	NFHS-3 (2005-06)	NFHS-4 (2015-16)	NFHS-5 (2019-21)
Early initiation of breastfeeding within one hour of birth (%)	15.8	24.5	24.5	41.8
Exclusive breastfeeding (zero to six months) (%)	41.2	46.4	54.9	63.7
Complementary feeding (six to nine months) (%)	35	56.7	42.7	45.9

Appropriate nutrition in early childhood pivots on embracing the practices, encircling breastfeeding and complementary feeding in cooperation. NFHS-5 reveals a decrease in early breastfeeding initiation in 12 states and Union Territories. There is a decreasing trend in early breastfeeding initiation observed in specific regions, including Sikkim, with a decline of 33.5%; Dadra and Nagar Haveli, with 24.1%; and Assam, with 15.3%. Contrastingly, Lakshadweep, Meghalaya, and Andhra Pradesh verified a striking surge in the rates of breastfeeding commencement. When comparing these data with NFHS-4 findings, an improvement is observed, although it may be characterized by marginal success. Moreover, the steep decrease in breastfeeding, which is done exclusively, declined by 26.3% in Sikkim. There was a declining trend in adopting the practice of the timely introduction of semisolid food to children in Himachal Pradesh and Kerala. Nevertheless, notwithstanding this trend, there was an extensive surge in the proportion of kids familiarized with complementary feeding on time, with over a 3% increase in the child undernutrition rate. In the timely introduction of complementary feeding, Tripura exhibited a notable increasing trend of 39.5% points [13,14].

The least standard diet serves as a composite indicator, considering the diversity and frequency of feeding in an individual's diet. Among the states, only Meghalaya has demonstrated improvement, with an increase of over 25%. Mizoram, Assam, Nagaland, Jammu and Kashmir, and Telangana have experienced a decreased trend in young children's overall proportions when provided with a sufficient diet. As per NFHS-5 data, trends in complementary feeding practices in Himachal Pradesh, Goa, Kerala, Nagaland, Tripura, and Telangana proved a surge of 3%. In addition, in Himachal Pradesh, Goa, and Kerala, the dietary competence remained almost the same. The decreasing trend in complementary feeding practices and diet adequacy between six and eight months was seen in Telangana and Nagaland, and increasing trends were seen in childhood stunting and overweight. Conversely, there was a decreasing trend of 5% in the stunting rate detected in Bihar. The encouraging trends of undernutrition in children in Manipur were projected toward a mutual impact of improvements in nutrition-sensitive indicators, including household profile, women's empowerment, pregnancy and delivery care, as well as the adequacy of diet and access to food. In Sikkim, there has been a considerable increase in the occurrence of overweight and obesity among females, rising from 26% in NFHS-4 to 34% in NFHS-5. Also, improvements were acknowledged as the rate of low height in children has improved. However, the trend of complementary feeding has slightly changed, changing from 23% in NFHS-4 to 24% in NFHS-5. There is an increasing trend in the percentage of children receiving complementary feeding within the recommended age of six to eight months. Alongside, there is a substantial decreasing trend in breastfeeding practices, declining from 54% in NFHS-4 to 28% in NFHS-5 in the state of Sikkim [13,14].

Discussion and challenges

India has made commendable development in promoting IYCF practices over the years. The government, in collaboration with various health organizations, has launched several programs like the National Nutrition Mission, Integrated Child Development Services (ICDS), and Pradhan Mantri Matru Vandana Yojana (PMMVY), which have significantly contributed to improving IYCF practices. These programs have created attention and counseling for lactating mothers, and strengthened the healthcare infrastructure. One of the achievements has been the growth in different breastfeeding trends, but despite that, various challenges and areas for improvement are still needed. Children frequently lack proper nutrition, health, and development, particularly in developing nations where undernutrition is one of the main causes of death for children under five. The overall health development of infants and young children is significantly impacted by the practices of the IYCF. In India, many factors influence these practices, encompassing cultural beliefs, socioeconomic status, and education in healthcare services access [11,15-20].

However, despite several efforts at breastfeeding techniques, the EBF rates in India remain low. Several factors contribute to this, including lack of information, inadequate assist structures, and the effect of formula milk marketing. Additionally, cultural practices, including the early introduction of water, honey, or different traditional meals called prelacteals, can preclude distinct breastfeeding. Complementary feeding practices in India also face demanding situations. The introduction of complementary meals has to begin around six months of age, similar to persistent breastfeeding. However, irrelevant and insufficient complementary feeding practices are prevalent, resulting in malnutrition and stunted growth in lots of children. Factors including restricted knowledge about appropriate meals, confined entry to nutritious meals, and terrible hygiene practices contribute to those challenges. Numerous initiatives have been applied in India to address these problems. The government has launched various services and health programs like ICDS to promote quality IYCF practices. These services aim to provide education and assistance to mothers, improve access to healthcare offerings, and improve the overall healthcare system. These services are provided by Anganwadi centers established mainly in rural areas and staffed with frontline workers. Under the Supplementary Nutrition Programme segment of the ICDS, children below six years of age and pregnant and lactating mothers are identified within the community. They are provided with supplementary feeding and growth monitoring services. The beneficiaries are given 300 days of supplementary feeding. The scheme tries to bridge the caloric gap between the national recommended and average intake of children and women in low-income categories by giving supplementary feeding. Furthermore, nongovernmental organizations (NGOs) and network-based total interventions have been running toward elevating the significance of breastfeeding and appropriate complementary feeding practices. These schemes focus on educating lactating mothers, families, and healthcare providers about the benefits of EBF, proper nutrition, and hygiene practices [21-31].

The large population of India offers a dynamic situation for exploring practices related to IYCF. In the Indian context, the point of interest in IYCF is paramount due to the large population of children under the age of five years. Adequate nutrition during this period is crucial for preventing malnutrition, helping cognitive development, and building a foundation for a healthy life. India's diverse cultural panorama brings a variety of traditional beliefs and practices associated with infant and younger baby feeding. These practices, deeply rooted in cultural norms, can substantially influence the selections made by mothers and caregivers [4,32-35].

Breastfeeding is historically valued in Indian society. There is an ancient and cultural significance attached to breastfeeding, frequently considered as an image of maternal care and nourishment. Efforts were made to promote EBF for the first six months and continue breastfeeding after six months with CF for up to two years and above. With ongoing urbanization and modifications in lifestyle, there is a shift in nutritional styles, mainly to demanding situations in keeping conventional IYCF practices. The availability and marketing of processed meals and the effect of working mothers on breastfeeding practices are areas of concern. The effectiveness of IYCF practices is intently related to the provision and accessibility of healthcare services. Adequate antenatal care and postnatal care, coupled with instructional interventions, contribute to the fulfillment of IYCF initiatives. Socioeconomic factors and education influence IYCF practices. Understanding the sociocultural, monetary, and healthcare contexts is critical for designing and imposing effective IYCF interventions in India. The situation is dynamic, with ongoing efforts to address challenges and improve fine practices. The alliance among government agencies, NGOs, and healthcare workers is important for ensuring the achievement of IYCF tasks and improving the nutritional status of India's youngest population [36,37].

Recommendations

Effective implementation of policies like the National Nutrition Mission (Prime Minister's Overarching Scheme for Holistic Nourishment Abhiyaan), ICDS, PMMVY, and the Infant Milk Substitutes Act is crucial. Ensuring stringent monitoring and evaluation can enhance the impact of these initiatives. Conducting mass media campaigns and community-based education programs can raise awareness about the importance of breastfeeding and appropriate complementary feeding practices. Training healthcare providers on IYCF counseling and establishing breastfeeding support groups within communities can support mothers. Encouraging workplace policies that support breastfeeding, such as maternity leave, breastfeeding breaks, and providing lactation rooms, can help working mothers continue breastfeeding. Involving community leaders and influencers in promoting optimal IYCF practices can help address cultural barriers and change traditional practices. Implementing social protection programs and providing food assistance to vulnerable families can ensure children receive adequate and nutritious complementary foods [38].

## Conclusions

The results from the NFHS surveys show both progress and challenges, as well as areas for improvement in feeding practices in India. There has been a positive trend in recent years in the number of babies being breastfed within the first hour of birth, as well as the percentage of infants who are exclusively breastfed for the first six months. However, there remains a need for concerted efforts to promote EBF and appropriate complementary feeding. Targeted interventions should prioritize the education of mothers, families, and healthcare providers regarding the significance of optimal IYCF practices. Addressing barriers such as insufficient knowledge, cultural beliefs, and inadequate support systems is crucial for enhancing these practices.
